# Measuring maternal mortality: An overview of opportunities and options for developing countries

**DOI:** 10.1186/1741-7015-6-12

**Published:** 2008-05-26

**Authors:** WJ Graham, S Ahmed, C Stanton, CL Abou-Zahr, OMR Campbell

**Affiliations:** 1 Department of Obstetrics and Gynaecology, University of Aberdeen, UK; 2 Immpact, University of Aberdeen, UK; 3 Department of Population, Family and Reproductive Health, The Johns Hopkins Bloomberg Shool of Public Health, Baltimore, MD, USA; 4 Health Metrics Network, World Health Organization, Geneva, Switzerland; 5 Department of Epidemiology and Population Health, London School of Hygiene and Tropical Medicine, London, UK

## Abstract

**Background:**

There is currently an unprecedented expressed need and demand for estimates of maternal mortality in developing countries. This has been stimulated in part by the creation of a Millennium Development Goal that will be judged partly on the basis of reductions in maternal mortality by 2015.

**Methods:**

Since the launch of the Safe Motherhood Initiative in 1987, new opportunities for data capture have arisen and new methods have been developed, tested and used. This paper provides a pragmatic overview of these methods and the optimal measurement strategies for different developing country contexts.

**Results:**

There are significant recent advances in the measurement of maternal mortality, yet also room for further improvement, particularly in assessing the magnitude and direction of biases and their implications for different data uses. Some of the innovations in measurement provide efficient mechanisms for gathering the requisite primary data at a reasonably low cost. No method, however, has zero costs. Investment is needed in measurement strategies for maternal mortality suited to the needs and resources of a country, and which also strengthen the technical capacity to generate and use credible estimates.

**Conclusion:**

Ownership of information is necessary for it to be acted upon: *what you count is what you do*. Difficulties with measurement must not be allowed to discourage efforts to reduce maternal mortality. Countries must be encouraged and enabled to count maternal deaths and act.

## Background

In 2000, 189 countries signed-up to improve maternal health as one of the eight Millennium Development Goals (MDGs). Progress towards this MDG-5 can be measured using a wide variety of indicators [1]. Government and donor commitments to maternal health can be monitored using financial indicators and policy approvals. Investment in maternal health programmes can be tracked by measuring inputs (such as midwifery training), outputs (such as the number of midwives posted) and processes (such as the uptake of skilled delivery care) [2]. These indicators are necessary for planning, implementing and monitoring initiatives to improve maternal health. However, there is also a need to show progress in terms of impact: reduced mortality, complications and disabilities, and improved health. In general, however, it is easier to track the inputs and outputs of a programme than its impact [3].

For developing countries without routine registration and medical certification of cause of death, measuring who dies and the cause of death is particularly difficult, and maternal mortality is no exception [4]. Currently, two-thirds of countries do not have the means to fully count or register their populations. Long-term efforts are needed to strengthen country capacities for comprehensive routine reporting of births and deaths. In the interim, measurement scientists have devised a range of alternative approaches that can help many countries without comprehensive vital statistics to generate estimates of mortality for various population sub-groups and causes. These alternatives approaches have evolved considerably over the last half-century [5]. Some are predominantly empirical approaches which rely on capturing new data on deaths, and others predominantly analytical, adjusting or modelling existing data on deaths and other related variables. Advances in the measurement of child mortality have been more marked than for adult mortality [6], although techniques for some cause-specific adult causes, such as HIV/AIDS [7], have improved in the last two decades.

Maternal mortality, a subset of adult female deaths, has also benefited from new or enhanced approaches for use in resource-poor countries [8]. While none of these is ideal compared with the gold standard of complete death registration, they do enable many countries to begin to establish the magnitude of the problem within their own borders. This article aims to raise awareness of the alternatives among all who commission and act upon information on maternal mortality. We summarize the main opportunities and options for generating empirical estimates, describe their evolution and evaluation, and propose optimal measurement strategies for different country contexts. It is timely to emphasize these opportunities for a number of reasons, and not just because one of the two indicators for MDG-5 is maternal mortality. First, to further help empower countries to measure maternal mortality and 'own' their national estimates. Second, to challenge the prevailing view of measurement stagnation [9]: that maternal mortality is too difficult or too expensive to measure. Third, to respond to the heightened need for health outcome data owing to results-based financing of maternity services in developing countries [10].

## Methods

### Laying-out the opportunities and options

We focus in this paper primarily on measuring the magnitude and trends in maternal mortality at national and major sub-national levels; Table 1 defines the key terms and indicators we use. We do not address approaches whose main purpose is to identify or improve interventions to prevent maternal deaths, such as quality of care audits or confidential enquiries [11]. Similarly, we do not discuss the various approaches and indicators which may act as proxy measures of maternal mortality, but which also provide essential information for monitoring programmes, such as the UN process indicators [12] and Unmet Obstetric Need [13]; these are reviewed in several recent papers [14-16]. Opportunities for measuring maternal mortality, as for other mortality outcomes, can be categorized according to cost, complexity, time involved, desired precision of the estimates or comparability over time. The intended utility of the estimates affects the required scope and accuracy of the information required, and the availability of resources affects the suitability of different options. In this paper we focus on a practical, non-specialist perspective to understanding the alternatives. Table 2 shows the two overriding questions that must be asked from the outset, and which lead to further practical considerations. The final choice of options to measure maternal mortality is often an iterative process, which involves making trade-offs between all of the considerations in Table 2.

**Table 1:** Principal definitions and measures of maternal mortality

**Pregnancy-related death **is *the death of a woman while pregnant or within 42 days of termination of pregnancy, irrespective of the cause of death*. This is a time-of-death definition.
**Maternal death **is *the death of a woman while pregnant or within 42 days of termination of pregnancy, irrespective of the duration and the site of the pregnancy, from any cause related to or aggravated by the pregnancy or its management but not from accidental or incidental causes*. This definition requires cause-of-death information in order to exclude incidental causes.

**Maternal mortality ratio (MMR**): number of maternal deaths during a given time period per 100,000 live births during the same time period.

**Maternal mortality rate**: number of maternal deaths in a given time period per 100,000 women of reproductive age, or woman-years of risk exposure, in the same time period.

**Lifetime risk of maternal death: **the probability of maternal death across a woman's reproductive life, usually expressed in terms of odds.

**Proportion of maternal deaths among female deaths (PMDF)**: maternal deaths as a proportion of all female deaths of reproductive age, usually defined as 15–49, in a given time period.

**Table 2:** Two key issues to clarify prior to measuring maternal mortality

**Why is the estimate needed?**
• To generate a broad estimate of the magnitude of the problem
• To identify detailed causes, differentials and determinants
• To identify differences in levels within a country
• To permit cross-country comparisons
• To enable regular monitoring of progress

**What resources are available?**
• Existing data sources or data-collection opportunities, for example routine civil registration, large multi-purpose surveys
• Human resources, for example technical skills to design a survey, or to manage, analyse and interpret data
• Field budget, for example funds available for new data collection
• Time available, for example estimate needed immediately, in 1–2 years time, or longer

**Figure 1:**
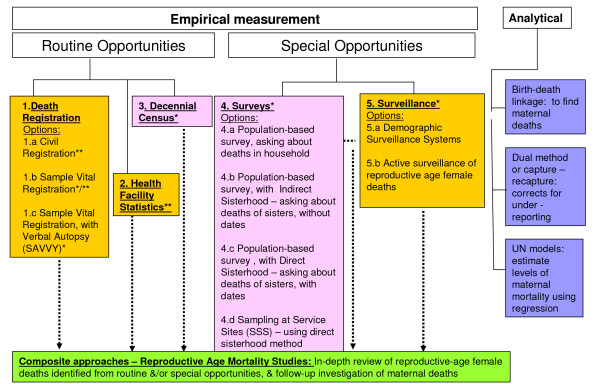
**Opportunities and options for measuring maternal mortality**. Colour key: Orange = longitudinal & continuous capture of deaths; Pink = cross-sectional capture; Green = mixed approach; Blue = no new capture of deaths. * Deaths actively sought by measurement option ** Deaths passively recorded, as dependent on relatives or health providers to notify death.

So what are these opportunities and options? Figure 1 introduces the alternatives schematically, and highlights the basic distinction between empirical approaches, the primary focus of this paper, and analytical approaches, which are discussed briefly later. Here we are also distinguishing between the primary mechanism or platform (measurement opportunity) for gathering the data, and the method (measurement option) used to identify maternal deaths and derive estimates of mortality. Figure 1 proposes five major data-gathering opportunities: (1) death registration; (2) health facilities; (3) decennial censuses; (4) surveys; and (5) surveillance. In addition, there are composite approaches which draw upon various combinations of these five to identify all deaths of women of reproductive age and then ascertain the maternal cases and circumstances. These are referred to collectively as Reproductive Age Mortality Studies (RAMOS) [17,18]. The five primary opportunities can be broadly grouped into routine or special sources. Generally speaking, routine opportunities yield a narrower range of information about maternal deaths than special studies but, with the exception of censuses, are continuously available and able to provide data for small geographical units. Moreover, as they form part of the wider information system, their use to measure maternal mortality involves minimal extra costs. The drawbacks, however, relate primarily to availability, reliability, completeness and coverage. Special studies, on the other hand, require more of the resources flagged in Table 2, but have the potential to produce detailed additional information on the circumstances of deaths. Their drawbacks relate primarily to margins of uncertainty due to both sampling and non-sampling errors, timeliness and predictability.

Figure 1 also shows that for some data-capture opportunities, such as surveys, there are a number of alternative methods or options that can be used within them to identify maternal deaths. For example, surveys provide an opportunity to apply methods which seek deaths reported in the household or among sisters, or deaths reported through respondents using sampling at service sites (SSS) [19], such as antenatal care. Additional file 1 acts as a reference resource, providing details on the characteristics of each option for measuring maternal mortality, as well as their strengths and limitations, and further supporting references [20]. There are also a number of web-based resources which provide additional details on specific options, now including a site dedicated to maternal mortality measurement [21].

Two other useful differences between opportunities for data capture are, firstly, whether deaths are identified actively or passively and, secondly, whether the starting point is all deaths, reproductive-aged female deaths, pregnancy-related deaths or maternal deaths (see Table 1 for definitions). Active identification enumerates cases in the population through direct interviews in the context of a census, survey or surveillance. Passive approaches, such as civil registration or health facility statistics, rely on deaths already captured by an existing system but go on to extract the maternal or pregnancy-related fraction. As a composite approach, RAMOS involves both active and passive identification of deaths. The distinction between active and passive is important in terms of completeness of reporting, with the latter more prone to omission of deaths and to bias because certain population subgroups are underrepresented.

The techniques for differentiating maternal from non-maternal deaths fall broadly into those with involvement from health professionals (medical certification or health facility data) versus those based on lay reports. With the involvement of health professionals the key distinction is between the presence or absence of diagnostic procedures, such as post-mortem, operative or laboratory results. With lay reports, maternal deaths are identified through time-of-death questions (yielding pregnancy-related mortality) or a verbal autopsy. The latter is a structured interview and/or narrative account administered to caregivers or family members of the deceased to identify signs and symptoms and thus determine probable cause(s) of death.

Alongside empirical measurement, there are three main analytical approaches which have been developed specifically for maternal mortality (Figure 1). Birth and Death Record Linkage identifies maternal deaths by using existing records of births (including stillbirths if available) and deaths obtained from routine civil registration or demographic surveillance. Records of births and reproductive-age female deaths are compared, and those which can be linked are deemed to be pregnancy-related deaths. Capture-recapture [22] or Dual (records) Methods use statistical methods to correct for underreporting from two sources of maternal deaths [23]. Finally, statistical models have been used to estimate levels of maternal mortality for countries without any reliable national-level empirical data. United Nations' [24] models use statistical regression and the proportion of maternal deaths among deaths of women of reproductive age to derive estimates of maternal mortality.

### Evolution and evaluation of opportunities and options

The five main empirical opportunities and related options for measuring maternal mortality (Figure 1) have evolved over last 20 years in response to the demand generated initially by the Safe Motherhood Initiative and sustained by the advent of MDG-5. In developing countries, heightened interest in data on maternal mortality began in the mid-1980s with a series of special studies, as discussed in a report by the WHO [25]. These revealed the serious underreporting in routine statistics and gave early insights into the challenges of capturing maternal deaths, particularly where the vast majority occurs without contact with the health system. Since then, considerably more experience has accumulated on these challenges and the published literature has been very explicit about them [26,27]; some would argue overly so [9]. The principal challenges are two-fold: first, to obtain sufficient or reliable detail, in official records or relatives' reports, to differentiate maternal from non-maternal causes; and, second, due to the comparative rarity of the event on a population basis, extremely large samples or complete enumeration are required to produce stable estimates. However, whilst facing similar difficulties to all-cause and cause-specific adult mortality, the maternal sub-group also has some positive features which make ascertainment easier and likely to be more complete. In most settings, pregnancy is a memorable event, and death related to pregnancy among otherwise healthy young women even more so. These deaths also cluster around the time of labour and delivery and in the following 24 hours, in other words at a time when the woman's pregnancy status is well recalled by reporting relatives [28].

Paying increased attention to the challenges of measuring maternal mortality has yielded benefits, both to the issue of maternal death itself and to the methods and empirical data available. For example, since national estimates for maternal mortality were first released in 1996 by WHO and UNICEF [24], the proportion of countries lacking usable data, and so dependent on modelled figures, has declined from almost half in 1990 to just over one-third in 2005 [29]. This growth in alternative measurement opportunities and options highlights the important contribution made by the Demographic and Health Surveys as a major platform for applying the Direct Sisterhood Method [30].

The different options for estimating maternal mortality detailed in Additional file 1 have different strengths and weaknesses. There is unfortunately no standard metric for valuing the advantages and disadvantages of measurement options. Rather comparisons need to be made based on broad categories and propensities for certain qualities. These are also summarized in Additional file 1. Inevitably, there are trade-offs to be made, for example between practical considerations, such as cost, time and statistical capacity, and scientific criteria of precision, reliability, comparability and validity. Many of the resource issues stem from the large sample sizes or complete enumeration needed, mentioned earlier, and this places practical and scientific considerations in direct competition. Moreover, the decision about size is not solely a statistical matter, but also influenced by the purpose of the resulting estimates, and hence the degree of certainty needed. There have been few publications on the comparative costs and benefits of different measurement opportunities and options. Generally speaking, routine and continuous systems, such as civil registration or demographic surveillance, are more cost-effective than special studies but require long-term commitment and attention to quality. Censuses are major undertakings, both in terms of human and financial resources, but the marginal cost of adding questions on maternal mortality is small [31].

From a scientific perspective, the validity and reliability of measurement options are of primary concern. There is, however, a conundrum with evaluating any new method for maternal mortality for use in low-income countries: the lack of existing estimates from a gold-standard source, namely, complete and accurate death registration. As a consequence, many so-called validation studies are, strictly speaking, comparative assessments of two or more alternatives, ideally applied to the same geographical area, population and time period. For example, recent work in Bangladesh compared pregnancy-related deaths in the household with deaths among sisters, and found extremely similar estimates of maternal mortality but with wide confidence intervals [8].

There are very few comprehensive appraisals of the magnitude of bias and uncertainty for the main opportunities and options for measuring maternal mortality. This gap needs to be addressed as a research and development priority, linking-up with similar efforts for other outcomes. One appraisal, of the Direct Sisterhood Method used by the DHS [32], found relative errors in the maternal mortality estimates averaging at 15% across 14 countries. Another form of appraisal has been published for the census as a means of measuring maternal mortality [31]. Although there are no sampling errors in such complete enumerations, non-sampling errors resulted in the need to upwardly adjust the numbers of adult female deaths, maternal deaths and, indeed, births in all but one of the five countries included in the assessment. In terms of health facility statistics, these will always be biased where some maternal deaths still occur in the community, but the direction of the error in estimates is hard to assess and quantify. For example, where facilities have a disproportionate fraction of high-risk deliveries, then the estimate may be much higher than that for the population as a whole and, conversely, where many deaths do not reach care, the facility figure will be an underestimate of the true population level. Moreover, this bias is often further aggravated by the omission of maternal deaths occurring on non-obstetric wards.

All opportunities and options for measuring maternal mortality in fact face the same two sources of error: identifying adult female deaths and/or determining whether such deaths are maternal or pregnancy-related. There are several demographic techniques (see Additional file 1) for assessing and adjusting identification of adult female deaths, although they cannot be used with sample surveys. Distinguishing whether deaths are pregnancy-related on the basis of time of death is widely regarded as valid in comparison with medical certification of death [33], although differentiating specific sub-causes of maternal death is more problematic. The recently developed standardized verbal autopsy tool developed by WHO represents a significant advance but will itself require validation [34]. Recent work with a computer-based algorithm [35] for assigning deaths from verbal autopsies which was applied in Burkina Faso showed that the Direct Sisterhood Method questions on time-of-death relative to pregnancy status differed by less than 10% from symptom-based questions in detecting pregnancy-related deaths.

### Country strategies for measuring maternal mortality

In this final section of the paper, we turn to the need for measurement strategies to generate estimates of levels and trends in maternal mortality, suited to specific contexts or developmental phases of a country. Additional file 2 proposes four phases in the evolution of measurement strategies, and provides examples of countries currently at these different stages. The phases are defined primarily on the basis of the status of the civil registration system and cause of death ascertainment, with complete and accurate coverage regarded as the optimum in the fourth and final phase. Even at this state of development, it is still relevant for other complementary measurement opportunities and options to be used, since no single approach can adequately meet all of the needs for information on maternal mortality.

As can be seen from Additional file 2, there is considerable overlap in strategies between phases. We emphasize the importance of taking advantage of add-on opportunities provided via the decennial census and large multi-purpose surveys, since the incremental costs of obtaining data on maternal mortality from these sources is marginal. However, these are not very timely, usually every decade for the census and every 4–5 years for large multi-purpose surveys, and often delayed in their implementation as well as the analysis and release of findings. For the four given states of the civil registration system in Additional file 2, other measurement opportunities are proposed on the basis of the likely resources available and the completeness and accuracy of health facility statistics. A practical guide on how to select between the different options for measuring maternal mortality shown earlier in Additional file 1 and for the specific phases indicated in Additional file 2 is now available as a web-based resource [21].

There are two further key requirements in developing countries for any measurement strategy to yield the reliable, timely and comparable data on maternal mortality required by decision-makers. First, the data must be processed, analysed, interpreted and communicated. This analysis should include estimation of uncertainty surrounding the maternal mortality indicators, as well as the use of various adjustment techniques to correct for under- or over-reporting of deaths or births. More developmental work is needed to refine some of these techniques, and to help reconcile and understand variation in estimates from different sources. Secondly, all measurement strategies depend on the skills and capacity of personnel in-country to undertake competently all stages from design through to communication, sometimes with external technical support. There is an urgent need to strengthen this skills base for all aspects of health information systems in developing countries [36].

## Conclusion

There is currently an unprecedented expressed need and demand for estimates of maternal mortality in developing countries. This has been stimulated in part by the creation of an MDG that will be judged partly on the basis of reductions in maternal mortality by 2015. The proposed shift towards results-based financing of maternal, neonatal and child health programmes by donors is now adding further incentives to improve data on this and other outcome indicators [37]. There are significant challenges to meeting these needs and demands which we would be foolish to ignore. Many of these are, however, very similar to the issues faced and, to a degree, overcome by other specific health problems, such as HIV/AIDS. The limitations of civil registration and routine health information systems in many countries are serious, but only by making maximum use of those data which are adequate and by investing in a continuous process of improvement will these be realized as the optimal sources [36]. Universal counting of maternal deaths should be the goal [38]. This aspiration does not, however, mean an indefinite wait for high-quality data on maternal mortality.

Since the launch of the Safe Motherhood Initiative in 1987, new opportunities for data capture have arisen and new methods have been developed, tested and used. No approach, however, can be perfect, and there is certainly still much room for improvement, especially assessing the magnitude and direction of biases and their implications for different data uses. Some of the innovations in measuring maternal mortality provide efficient mechanisms for gathering the requisite primary data at a reasonably low cost. No method, however, has zero costs. Investment is needed in measurement strategies for maternal mortality suited to the needs and resources of a country, and which also strengthen technical capacity to generate and use credible estimates. Ownership of information is necessary for it to be acted upon: *what you count is what you do *[39]. Difficulties with measurement must not be allowed to discourage efforts to reduce maternal mortality. Countries must be encouraged and enabled to count maternal deaths and act.

## Competing interests

The authors declare that they have no competing interests.

## Authors' contributions

WJG led the conceptualization and design of the paper, and the drafting, revisions and finalization of the article. SA, CS, CAZ participated in the drafting, revisions and finalization of the paper. OMC contributed to the design of the paper, prepared Additional File 1, and participated in the drafting, revisions and finalization of the paper. All authors read and approved the final manuscript.

## Pre-publication history

The pre-publication history for this paper can be accessed here:



## Supplementary Material

Additional file 1 Typology of opportunities and options for measuring maternal mortality [40-48]. Click here for file

Additional file 2 Phases of country measurement strategies for maternal mortality [29]. Click here for file
