# Supplementary material for: Measuring maternal mortality: An overview of opportunities and options for developing countries
Source: BMC Med. 2008 May 26;6:12. doi: 10.1186/1741-7015-6-12 (PMC2430703; doi:10.1186/1741-7015-6-12)
Supplement: Additional file 1 — Typology of opportunities and options for measuring maternal mortality [40-48]. [file 1741-7015-6-12-S1.doc]

**TABLE 1 TYPOLOGY OF OPPORTUNITIES AND OPTIONS FOR MEASURING MATERNAL MORTALITY**

| **Data collection opportunities** | **Option for identifying maternal deaths** | **Strengths and limitations** |
| --- | --- | --- |
| **1 DEATH REGISTRATION**  **1a. Civil Registration**:  The UN defines this as: “the continuous, permanent, compulsory and universal recording of the occurrence and characteristics of vital events (live births, deaths, foetal deaths, marriages and divorces).”  1b. **Sample Registration** (SRS): Involves registration of births and deaths in nationally-representative sample of areas, but invokes no legal issues such as certification.  1c. **Sample Vital Registration with Verbal Autopsy** (SAVVY) [ 40] | - Family member and/or health facility notify deaths to civil registration system - Cause of death medically certified according to ICD [ 41] - Modified Death Notification Form [ 42] can be used to flag if death is pregnancy-related - For SAVVY, lay reporting of cause of death based on signs and symptoms or verbal autopsy | **Strengths**:   - Serves multiple purposes; is representative of entire population; does not require special data collection activities; continuously available. - Provides annual & cause-specific estimates of maternal mortality, trends & regional differentials; also provides data on births.   **Limitations:**   - Under-reporting of deaths needs to be assessed/adjusted using demographic techniques [ 43,44]. - Quality of cause-of-death certification and coding needs regular assessment. - Untimely/late reporting of deaths and births. - SRS & SAVVY: does not include formal certification. - SAVVY: Generally requires active follow up and household visits to determine cause of death & quality of cause-of-death reporting may be problematic. |
| **2. HEALTH FACILITY STATISTICS**  Facilities compile statistics on maternal deaths & births, & report these to the central level | - Facility records deaths - Medical professional gives ICD cause - May use diagnostic aids or autopsy (post-mortem) | **Strengths:**   - On-going systems that do not require special data collection activities - Provides annual & cause-specific estimates of maternal mortality and trends   **Limitations:**   - Only captures facility deaths & prone to miss those occurring in non-obstetric wards [ 45] - Records may be poorly maintained; chain of moving data to central level may breakdown |
| **3. DECENNIAL CENSUS** [ 31]  The process of collecting, compiling, evaluating, analysing & disseminating demographic, economic & social data pertaining, at a specified time, to all persons in a country or in a well-delimited part of a country. | - Adult household member reports retrospectively all births and deaths of household members (& age & sex) within a specified period, usually 1 or 2 years [ 31]. (Called “direct questions”) - Adult reports if death of an adult female aged 15-49 is pregnancy-related (time-of-death relative to pregnancy, childbirth and postpartum period). | **Strengths:**   - Provides birth & death data for entire population. - Enables recent estimates of adult & pregnancy-related mortality for small areas; provides data on socio-economic characteristics of households with maternal deaths. - Lengthens the duration of the census interview only negligibly.   **Limitations:**   - Only usually occurs every 10 years, & analysis/reporting often delayed. - Disintegration of household after death can mean there is no “household” to report. - No standard measures for defining uncertainty due to non-sampling errors. - Under/over reporting of births and deaths need to be adjusted using demographic techniques [ 44]. |
| **4. SURVEYS:**  **4a.Population-based household surveys with direct estimation:** sampling methods ensure estimates represent the population | - Deaths identified via direct mortality questions described above for the census - Adult responds to pregnancy-related questions or verbal autopsy [34 ] to identify maternal deaths | **Strengths:**   - Provides birth & death data for population sampled. - Enables recent estimates of adult & pregnancy-related mortality, & data on determinants. - Confidence intervals can be calculated.   **Limitations**:   - Requires a large sample to capture enough maternal deaths for reliable estimates. - Costly for monitoring trends & problematic as overlapping confidence intervals. - Household disintegration after death may mean there is no “household” to report. - Deaths usually under-reported (but may be over-reported); demographic adjustment techniques for mortality cannot be used in sample surveys. |

| **Data collection opportunities** | **Option for identifying maternal deaths** | **Strengths and limitations** |
| --- | --- | --- |
| **4b.Population-based household surveys with Indirect Sisterhood method** [ 46] | - Adult respondent reports death of his/her adult sisters retrospectively - Adult responds to pregnancy-related questions on sisters’ deaths aged 15-49 to identify maternal deaths | **Strengths:**   - Provides birth & death data representative of population - Adds only 4 questions to survey; easy to add to multipurpose surveys; simple data processing & calculation of estimates; permits calculation of confidence intervals   **Limitations**:   - Assumes unchanging fertility, & less appropriate where substantial migration. - Gives retrospective estimates (usually for 10-12 years before the survey). - Generally has large confidence intervals (as a function of reduced sample size) - No established demographic techniques to permit evaluation and adjustment, if necessary |
| **4c.Population-based household surveys with Direct Sisterhood Method**  30] | - Adult respondent reports death (and sex and age at death) of his/her adult siblings retrospectively to interviewer - Adult responds to pregnancy-related questions or verbal autopsy [ 33] on sisters’ deaths aged 15-49 to identify maternal deaths | **Strengths:**   - Provides data that represent the population; - Generates a more recent estimate than indirect method; permits calculation of confidence intervals; can be added to multipurpose surveys.   **Limitations**:   - Larger sample size than indirect method, & more complex collection, processing & analysis. - Less appropriate where substantial migration. - No established demographic techniques to permit evaluation and adjustment, if necessary. |
| **4d. Sampling at Service Sites (SSS)** [ 19]  this uses non-probability sampling by gathering data opportunistically at service sites (antenatal or child health services, or market places), but uses high coverage sites and data on characteristics of the sampled population to assess biases. | - Adult respondent reports death using Direct Sisterhood method (see above) | **Strengths:**   - Cost-efficient means of greatly increasing sample size, & thus more frequent measurement. - Requires less time for data collection & analysis. - Shares other strengths as Direct Sisterhood Method, described above.   **Limitations**:   - Potential selection biases in respondents needs to be assessed. - Interview must be reasonably short & thus limited other data can be gathered. - Incentives may be needed for respondents in markets. - Shares other limitations as Direct Sisterhood method, described above. |
| **5.SURVEILLANCE OPTIONS**  **5a. Demographic Surveillance Sites (DSS)** [ 47]**:** DSS are usually larger than prospective maternal mortality studies and measure a range of demographic endpoints. | - Enumerators make initial census/count of population - Adult household member reports deaths since last round - Occasionally DSS is coupled with active reporting of deaths by key informants (see row below) | **Strengths:**   - More complete enumeration than Civil Registration in countries with weak infrastructure. - Provides comparable data over time for trends, & researchers for data analysis.   **Limitations**:   - Costly and research intensive. - May cover relatively small portion of country, and not nationally representative. - Women may migrate out or into DSS area for delivery. |
| **5b. Active surveillance of reproductive age female deaths, using key informants:**  Local key informants may include TBAs, health care providers, village leaders, teachers, cemetery workers. | - Key informants actively report deaths to women of reproductive age & pregnancy-related deaths, & sometimes live births too - Households may be visited for verbal autopsy interview to confirm maternal deaths [ 48]. | **Strengths:**   - Allows data collection using existing personnel resources, thus cost-efficient.   **Limitations**:   - Maintaining quality of reporting may be difficult in longer-term. - Accuracy is improved if two networks of informant are used and capture/recapture techniques used to adjust for underreporting. |
