# Supplementary material for: Measuring maternal mortality: An overview of opportunities and options for developing countries
Source: BMC Med. 2008 May 26;6:12. doi: 10.1186/1741-7015-6-12 (PMC2430703; doi:10.1186/1741-7015-6-12)
Supplement: Additional file 2 — Phases of country measurement strategies for maternal mortality [29]. [file 1741-7015-6-12-S2.doc]

**TABLE 2 PHASES OF COUNTRY MEASUREMENT STRATEGIES FOR MATERNAL MORTALITY**

| **PHASE** |  | **STATUS OF CIVIL REGISTRATION** |  | **MEASUREMENT STRATEGY** |  | **ILLUSTRATIVE EXAMPLES OF COUNTRIES^** |
| --- | --- | --- | --- | --- | --- | --- |
| **I** |  | No registration of births or deaths, and no medical certification of cause of death. |  | - Take advantage of Census and multi-purpose surveys to measure pregnancy-related deaths at national level. Census may provide opportunity to conduct follow-up verbal autopsies for representative areas. - If funds* available, implement RAMOS in representative sample of areas, or a large maternal mortality survey. - Train medical practitioners in cause of death certification for deaths that occur in health facilities and ensure that death certificates include pregnancy checkbox. - If funds limited, use SSS in representative sample of areas, with follow-up verbal autopsies involving community-based health professionals. - Use model-based estimates as additional source of estimate for country-specific national estimates. - Start to implement registration of births and deaths in sample areas, and mobilize communities to support reporting. - If large demographic surveillance sites exist, ensure maternal deaths are reported and use for gauging sub-national levels. |  | Afghanistan, Burkina Faso, Ethiopia, Malawi, Nepal, Uganda |
|  |  |  |  |  |  |  |
| II |  | Incomplete or patchy registration of births and deaths, and limited medical certification. |  | - Take advantage of Census and multi-purpose surveys to measure pregnancy-related deaths at national level. Census may provide opportunity to conduct follow-up verbal autopsies for representative areas. - If major funds available, implement RAMOS in representative sample of areas, or a large maternal mortality survey. - Train medical practitioners in cause of death certification for deaths that occur in health facilities and ensure that death certificates include pregnancy checkbox - If funds limited, use SSS in representative sample of areas with follow-up verbal autopsies involving community-based health professionals. - Use model-based estimates as additional source of estimate for country-specific national estimates. - Strengthen and extend registration areas, alongside improvements in routine health information system, especially in urban areas, and mobilize communities to support reporting. |  | Cambodia, Egypt, India, Mauritania, Myanmar, Thailand |
|  |  |  |  |  |  |  |
| III |  | Countries with complete (>90%) birth and death registration, but inadequate medical certification. |  | - Focus on strengthening certification by training medical and statistical personnel. - If high proportion of deaths occur in or have contact with the health system, use routine health information system and periodic CEMD to confirm cause of death pattern. - If funds* available, implement national RAMOS. - If funds limited, use SSS with follow-up verbal autopsies involving community-based health professionals, for specific high mortality/problematic areas. - Train medical practitioners in cause of death certification for deaths that occur in health facilities and ensure that death certificates include pregnancy checkbox. |  | Argentina, Colombia, Greece, Poland, Qatar,  South Africa |
| IV |  | Countries with complete birth and death registration, and good medical certification |  | - Ensure quality of cause of death certification is sustained by comparison with data from routine health information system, periodic CEMD or special surveillance - Using analytic methods to confirm overall completeness of death registration - If funds limited, use SSS with follow-up verbal autopsies involving community-based health professionals, for specific high mortality/problematic areas. |  | Australia, Bahamas, Costa Rica, Israel, Mexico, Uzbekistan |

* Funds for data capture and technical support

^ Based on estimation exercise [29]
